# Histone Deacetylase 11 Knockdown Blocks Larval Development and Metamorphosis in the Red Flour Beetle, *Tribolium castaneum*

**DOI:** 10.3389/fgene.2020.00683

**Published:** 2020-07-03

**Authors:** Smitha George, Subba Reddy Palli

**Affiliations:** Department of Entomology, University of Kentucky, Lexington, KY, United States

**Keywords:** juvenile hormone, epigenetics, histone deacetylase 11, histone H3, dsRNA

## Abstract

Post-translational modifications (PTM) such as methylation, acetylation, phosphorylation, and ubiquitination of histones and other proteins regulate expression of genes. The acetylation levels of these proteins are determined by the balance of expression of histone acetyltransferase (HATs) and histone deacetylases (HDACs). We recently reported that class I HDACs (HDAC1 and HDAC3) play important roles in juvenile hormone (JH) suppression of metamorphosis in the red flour beetle, *Tribolium castaneum*. Here, we report on the function of a single class IV HDAC member, HDAC11. Injection of dsRNA targeting *T. castaneum HDAC11* gene into newly molted last instar larvae induced knockdown of the target gene and arrested larval development and prevented metamorphosis into the pupal stage. Dark melanized areas were detected in larvae that showed developmental arrest and mortality. Developmental expression studies showed an increase in HDAC11 mRNA levels beginning at the end of the penultimate larval stage. These higher levels were maintained during the final instar larval and pupal stages. A JH analog, hydroprene, suppressed *HDAC11* expression in the larvae. Sequencing of RNA isolated from control and dsHDAC11 injected larvae identified several differentially expressed genes, including those involved in JH action, ecdysone response, and melanization. The acetylation levels of core histones showed an increase in TcA cells exposed to dsHDAC11. Also, an increase in histone H3 acetylation, specifically H3K9, H3K18 and H3K27, were detected in HDAC11 knockdown larvae. These studies report the function of HDAC11 in insects other than *Drosophila* for the first time and show that HDAC11 influences the acetylation levels of histones and expression of multiple genes involved in *T. castaneum* larval development.

## Introduction

Two major insect hormones, ecdysteroids (20-hydroxyecdysone, 20E, is the most active form) and juvenile hormones (JH) regulate many aspects of insect life, including postembryonic development (Riddiford, 1996; Wyatt et al., 1996; Hartfelder, 2000; Singtripop et al., 2000; Min et al., 2004; Giray et al., 2005; Hrdy et al., 2006; Futahashi and Fujiwara, 2008). These hormones have been extensively studied because of their involvement in the regulation of multiple biological processes, including diapause, reproduction, and polyphenism (Wigglesworth, 1934; Riddiford, 1994, 2012). Juvenile hormones are sesquiterpenoids secreted by the corpora allata that mediate a variety of functions in insects (Jindra et al., 2013). The JH signaling cascade is a complex molecular process that includes multiple players such as JH receptor, *Methoprene-tolerant* (*Met*) (Wilson and Fabian, 1986; Konopova and Jindra, 2007) and, its heterodimeric partner, SRC, steroid receptor co-activator homolog (Li et al., 2011; Zhang et al., 2011). The JH-receptor complex binds to the juvenile hormone response elements (JHRE) present in the promoters of JH-response genes and regulate their expression (Kayukawa et al., 2012; Cui et al., 2014). 20-hydroxyecdysone binds to a heterodimer of two nuclear receptors, ecdysone receptor (EcR) and ultraspiracle (USP) and ecdysone response elements present in the promoters of ecdysone-induced transcription factors including E75, E74, Broad complex (BR-C) and E93 and regulate their expression (Palli et al., 2005). These ecdysone induced transcription factors, in turn, regulate expression of multiple genes important for growth, development, molting and metamorphosis (Riddiford et al., 2000).

Hormones represent attractive targets for the development of environmentally friendly insect control methods. Hindering this effort is the lack of knowledge on the molecular basis of hormone action. Research in epigenetics-based gene regulation has facilitated the discovery of various post-translational modification mechanisms (PTM) such as methylation, acetylation, phosphorylation, and ubiquitination. The role of acetylation in the regulation of 20E induced gene expression in *Drosophila melanogaster* has been reported (Bodai et al., 2012). The CREB-binding protein (CBP) mediates acetylation of histone H3K27 and antagonizes “Polycomb” silencing in *D. melanogaster* (Tie et al., 2009). The CBP also functions in regulating the expression of hormone response genes in *Tribolium castaneum* (Roy et al., 2017; Xu et al., 2018) and *Blattella germanica* (Fernandez-Nicolas and Belles, 2016). Since acetylation is a key component in the regulation of gene expression, we decided to explore the function of histone deacetylases (HDACs) in the red flour beetle, *T. castaneum.* Recent findings from our lab have demonstrated that class I HDACs (HDAC1 and HDAC3) play important roles in JH suppression of metamorphosis in *T. castaneum* (George et al., 2019; George and Palli, 2020). Here, we focused on the function of sole class IV HDAC member, HDAC11 (TC007473), to study its role in *T. castaneum* development.

Human HDACs identified to date can be grouped into four classes; Class I-IV based on their structure, phylogeny, and function. Class I HDACs are ubiquitously expressed and play essential roles in proliferation, whereas classes II and IV have a tissue-specific function (Lehrmann et al., 2002). HDAC11 first described in 2002 is a unique member class IV HDAC family since it is not homologous with RPD3 or HDA1 yeast enzymes (Gao et al., 2002). Selective/class-specific inhibitors targeting HDAC11 have been developed for treating patients with myeloproliferative neoplasms (MPN) (Yue et al., 2020). HDAC11 shows some sequence similarity to class I and II HDACs and is highly conserved in invertebrates and plants (Yang and Seto, 2008). HDAC11 depletion in neuroblastoma cell lines induces cell death mediated by apoptotic programs (Thole et al., 2017). HDAC11 knockout study in mice identified an age-dependent brain region-specific function in regulating *FEZ1* (fasciculation and elongation protein zeta 1), a gene associated with schizophrenia (Bryant et al., 2017). HDAC11 knockout mice showed resistance to high-fat-diet-induced obesity and metabolic syndrome, suggesting that HDAC11 functions as a critical metabolic regulator (Sun et al., 2018). However, not much information is available on HDAC11 function in insects. Functions of *D. melanogaster* histone deacetylases were studied by RNA interference and microarrays and showed that HDAC1 and HDAC3 control expression of genes involved in multiple processes including lipid metabolism, cell cycle regulation and signal transduction (Foglietti et al., 2006). However, three other HDACs tested did not show any detectable functions (Foglietti et al., 2006). Also, overexpression of HDAC 3, 6 or 11 suppressed CGG repeat-induced neurodegeneration in *D. melanogaster* Fragile X Tremor Ataxia Syndrome model suggesting that HDAC activators might be used to repress transcription of fragile X syndrome gene (Todd et al., 2010). In the current studies, we employed RNAi, RNA sequencing, and RT-qPCR to elucidate the role of HDAC11 in *T. castaneum*. Knockdown of HDAC11 during the larval stage induced arrest in larval development, melanization, and mortality. RNA isolated from *T. castaneum* larvae injected with double-stranded RNA (dsRNA) targeting the gene coding for HDAC11 (dsHDAC11) or dsmalE (a control dsRNA targeting *E. coli* malE gene) was sequenced, and differential gene expression analysis was conducted. Genes involved in hormone action and multiple biological processes such as melanization were identified as differentially expressed genes in HDAC11 knockdown larvae.

## Materials and Methods

### Insects and Cells

Insects (*T. castaneum*, GA-1 strain) (Haliscak and Beeman, 1983) were maintained in a Percival incubator set at 30°C and 65 ± 5% relative humidity with complete dark conditions on organic wheat flour (Heartland Mill, Marienthal, KS) mixed with 10% baker’s yeast (MP biomedicals, Solon, OH, United States). The *T. castaneum* cells, BCIRL-TcA-CLG1 (TcA), were cultured in EX-CELL 420 (Sigma-Aldrich, St-Louis, MO, United States) medium supplemented with 10% Fetal Bovine Serum (FBS, VWR-Seradigm, Radnor, PA, United States) at 28°C as described previously (Goodman et al., 2012).

### Hormone Treatments

Both *S*-Hydroprene (Ethyl 3, 7, 11-trimethyl-2, 4-dodecadienoate) and JH III were purchased from Sigma-Aldrich. The hydroprene was dissolved in cyclohexane at 2 μg/μl concentration and one microliter of this solution was applied on the integument of each last instar larva. One microliter of cyclohexane solvent alone was applied to each control larva. For cell culture experiments, JH III was prepared in DMSO at 10 mM concentration and one microliter per ml of culture medium was added to achieve a final concentration of 10 μM. Control cells were treated with the same volume of DMSO.

### Double-Stranded RNA Synthesis (dsRNA), RNA Isolation, cDNA Synthesis and Quantitative Reverse Transcription PCR (RT-qPCR)

*T. castaneum* HDAC11 ortholog was identified using the *D. melanogaster* HDAC11 sequence available at the FlyBase (Thurmond et al., 2019). Specific primers targeting two regions of *HDAC11* and containing T7 polymerase promoter at the 5′ end (Supplementary Table S1) and genomic DNA isolated from *T. castaneum* were used to PCR amplify the fragment of HDAC11. The PCR fragment used as a template for dsRNA synthesis. Primers used to amplify a fragment of JH receptor, Met, from *T. castaneum* (TcMet) have been reported previously (Parthasarathy et al., 2008). The MEGAscript T7 kit (Invitrogen, United States) was used for dsRNA synthesis. Purification, quality check, and quantification of dsRNA were performed as described previously (George et al., 2019). dsRNA prepared using a fragment of *Escherichia coli* maltose-binding proteins (malE) was used as a control.

Total RNA was isolated from treated and control insects using TRI reagent-RT (Molecular Research Center, Inc. Cincinnati, OH, United States). The RNA was converted to cDNA using M-MLV reverse transcriptase (Invitrogen-ThermoFisher Scientific). RT-qPCR was performed using iTaq Universal SYBR Green Supermix (Bio-Rad, Hercules, CA, United States) in Applied Biosystems StepOnePlus Real-time PCR instrument. The qPCR mixture contained 2 μl of diluted cDNA (1:5), 0.4 μl gene-specific primer mix, 2.6 μl nuclease-free water, and 5 μl SYBR green in a 10 μl final volume. The qPCR cycling conditions were: initial holding stage 95°C (20 s), followed by 40 cycles of denaturation at 95°C (5 s), annealing, and extension at 60°C (30 s) along with melting curve. The relative mRNA levels were calculated using *RP49* as a reference gene.

### Differential Gene Expression Analysis

Total RNA extracted from three biological replicates of larvae at 12 h after treatment with dsHDAC11 or dsmalE using the TRI reagent-RT and used for RNA-seq library preparation following the protocol described previously (Ma et al., 2014; Hunt, 2015; Kalsi and Palli, 2017). Raw reads were analyzed following CLC genomic workbench pipeline (Version 11.0.1, Qiagen, United States). Blast2GO Pro Plugin in the CLC workbench was used to determine the GO terms. The GO terms were used for predicting functions of differentially expressed genes. The GO terms were represented using Web Gene Ontology Annotation Plot (WEGO) as described previously (Ye et al., 2006).

### Western Blot Analysis

We used Acetylated-Lysine (Ac-K^2^-100) MultiMab^TM^ Rabbit mAb mix (Cell signaling #9814) to detect proteins post-translationally modified by acetylation. Histone H3 antibody sampler kit #9927 (Cell Signaling, Danvers, MA, United States) that includes Lys9, Lys14, Lys18, Lys27, and Lys56 specific antibodies were used to detect various lysine acetylation sites of histone H3. Band density was determined by Image-J software and normalized with loading control protein, ß-Actin. The Anti-rabbit IgG, HRP-linked antibody (Cell signaling #7074), was used for chemiluminescence detection. The blots were developed with Supersignal^TM^ West Femto Maximum sensitivity Substrate (Thermo Fisher, Scientific, Rockford, IL, United States).

### Statistical Analysis

The statistical analyses were performed using JMP Pro 14.0 (SAS Institute Inc., Cary, NC, United States) to test for statistical differences among treatments. *Post-hoc* tests were conducted using the Tukey-Kramer HSD method (α = 0.05). One-way ANOVA was performed for comparison between treatments.

## Results

### *Tribolium castaneum* HDAC11

**FIGURE 1:**
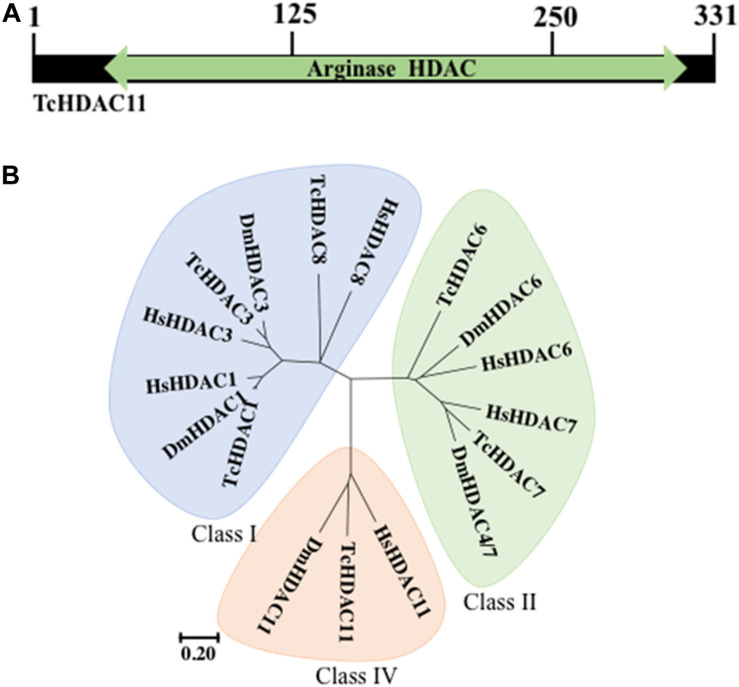
TcHDAC11 catalytic domain and phylogeny. **(A)** Schematic representation of the TcHDAC11 with the catalytic domain marked. The numbers on the top show the position of amino acids. The data for this Figure is obtained from NCBI (Geer et al., 2002). **(B)** Phylogenetic tree demonstrating the relationship between HDAC classes. The tree was inferred using the Neighbor-joining method. Evolutionary analyses were conducted in MEGA7 (Kumar et al., 2016). The tree was drawn to scale, with branch lengths and the evolutionary distances used to infer the phylogenetic tree as the same units. The identification of HDACs in *T. castaneum* was reported previously (George et al., 2019).

*T. castaneum* HDAC11 contains a single Zn+ dependent catalytic domain surrounded by a short N- and C- terminus (Figure 1A). Phylogenetic analysis of HDACs in *T. castaneum* revealed that TcHDAC11 is closer to Class I deacetylases (HDAC1, HDAC3, and HDAC8) than to class II (Figure 1B). *T. castaneum* HDAC11 open reading frame consists of 314–331 residues with a molecular mass of 35.2–37.2 kDa and one catalytic domain (Figure 1A). Comparison between the *T. castaneum* full-length HDAC11 amino acid sequence with those from *D. melanogaster* and human HDAC11s amino acid sequences showed 54 and 58 percent amino acid identity, respectively (Supplementary Figure S1).

### *HDAC11* Knockdown Arrests Larval Development

**FIGURE 2:**
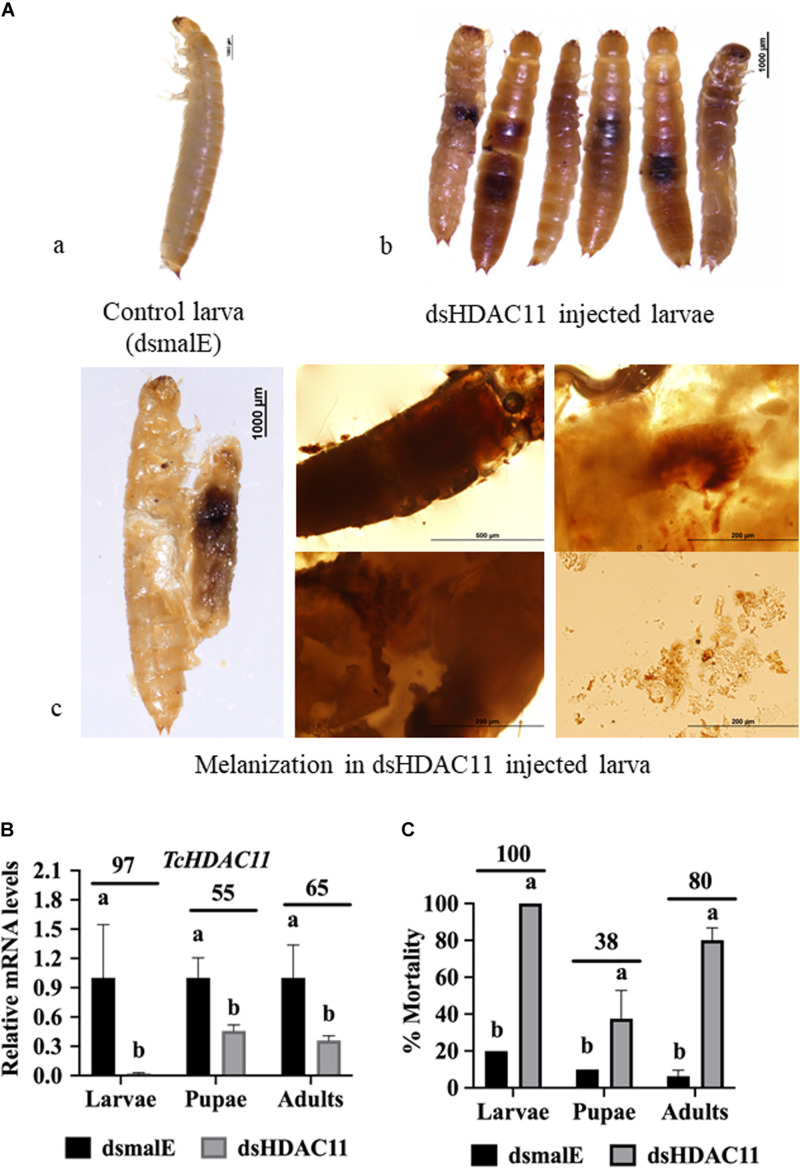
Phenotypes and mortality induced by dsRNA mediated knockdown of *HDAC11* in *T. castaneum*. **(A) a:** Control larvae injected with dsmalE pupated and later emerged as healthy adults. **b:** dsHDAC11 was injected into the newly molted last instar larvae. Phenotypes were photographed on the fifth day after injection. Knockdown of the *HDAC11* gene affected larval development resulting in pigmentation, growth retardation, and mortality. **c:** Light microscopic images of melanization in HDAC11 knockdown larvae. A high degree of hard melanization was detected inside the body. **(B)** Newly molted last instar larvae, pupae or adults were injected with dsHDAC11 or dsmalE. Total RNA was isolated from treated insects at 72 h after injection and used to determine relative mRNA levels of TcHDAC11. Knockdown efficiency was calculated by comparing TcHDAC11 mRNA levels in dsTcHDAC11 and dsmalE treated insects. Mean ± SE (*n* = 3–5) are shown. Means marked with different letters are significantly different from each other, *P* ≤ 0.05 by ANOVA. **(C)** Newly molted last instar larvae, pupae or adults were injected with dsHDAC11 or dsmalE. The mortality was recorded until death or adult eclosion. Mean ± SE (*n* = 30) are shown. Means marked with different letters are significantly different from each other, *P* ≤ 0.05 by ANOVA.

To determine HDAC11 function in larval, pupal development, and metamorphosis, the dsHDAC11 was injected into newly molted last instar larvae (day 0), freshly formed white-colored pupae and adults. The control insects injected with dsmalE (Figure 2Aa) pupated after 5–6 days after dsRNA injection and later emerged as healthy adults. However, developmental arrest and mortality were observed in 100% of dsHDAC11 injected larvae (Figure 2Ab). The arrested larvae showed dark pigmentation inside their body (Figure 2Ac). Dark melanized patches of tissues attached to the integument were detected in dissected larvae. Injection of dsHDAC11 reduced mRNA levels of the target gene by 97, 55, and 65% in larvae, pupae and adults, respectively, when compared to their levels in control insects injected with dsmalE (Figure 2B). Injection of dsHDAC11 also induced 100, 38, and 80% mortality in larvae, pupae and adults, respectively (Figure 2C). In contrast, the control insects injected with dsmalE showed less than 20% mortality (Figure 2C).

### Developmental Expression and JH Suppression of TcHDAC11

**FIGURE 3:**
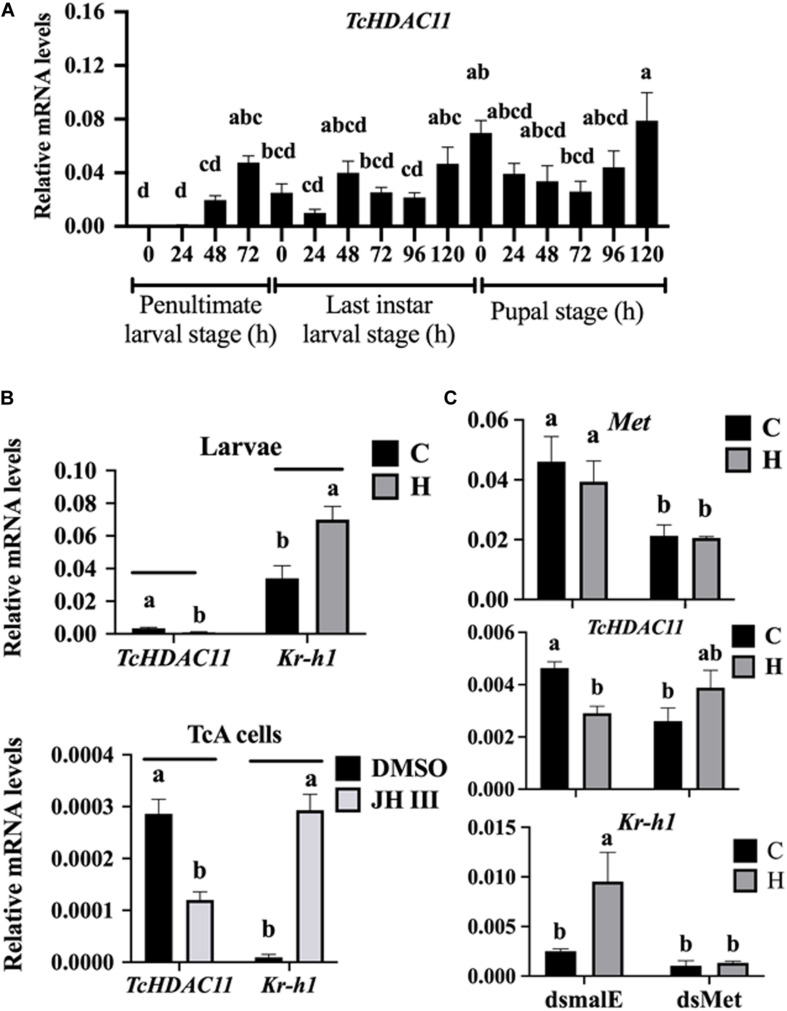
Developmental expression profile and JH induction of *HDAC11* in *T. castaneum* determined by RT-qPCR. **(A)** HDAC11 mRNA levels were determined during the penultimate, last larval, and pupal stages at 24 h intervals. Total RNA was isolated from a pool of two larvae for each replication and subjected to RT-qPCR analysis to determine the relative mRNA levels. The HDAC11 mRNA levels were normalized using RP49. Results expressed as Mean ± SE (*n* = 4). Means marked with different letters are significantly different from each other, *P* ≤ 0.05 by ANOVA. **(B)** JH suppresses the expression of *HDAC11* in *T. castaneum* larvae and TcA cells. S-Hydroprene (JH analog) dissolved in cyclohexane was topically applied to 48 h old last instar larvae (0.5 μL of 2 μg/μL). Total RNA was isolated from larvae collected at 6 h after treatment and subjected to RT-qPCR. Similarly, TcA cells were treated with 10 μm of JH III in DMSO or DMSO alone for 6 h. Total RNA isolated from larvae was used to quantify Kr-h1 and HDAC11 mRNA levels. Mean ± SE (*n* = 4) are shown. **(C)** Met is required for suppression of *HDAC11* by hydroprene. dsMet or dsmalE was injected into day 0 last instar larvae. At 48 h after injection, the larvae were treated with hydroprene. Total RNA isolated from larvae was used to quantify *Kr-h1*, *HDAC11* and, *Met* mRNA levels. The data shown are mean ± SE (*n* = 4). C, cyclohexane; H, hydroprene.

No HDAC11 mRNA was detected in *T. castaneum* larvae soon after molting into the penultimate larval stage (Figure 3A). Then the HDAC11 mRNA levels increased gradually, reaching the maximum levels by the end of the penultimate larval stage (Figure 3A). The HDAC11 mRNA was detected throughout the last instar larval and pupal stages, albeit with some fluctuations in their levels (Figure 3A). In general, higher levels of HDAC11 mRNA were detected at the end of the penultimate and last instar larval stages when compared to those at the beginning of these stages. Also, higher levels of HDAC11 mRNA were detected in the pupae when compared to those in the larval stages.

To test if JH suppresses HDAC11 gene expression, the last instar larvae were treated with JH analog hydroprene in cyclohexane or cyclohexane alone. As shown in Figure 3B, significantly lower levels of HDAC11 mRNA were detected in larvae treated with hydroprene when compared to those in the control larvae treated with cyclohexane. Similarly, lower levels of HDAC11 mRNA were detected in TcA cells exposed to JH III, when compared to those in the control cells exposed to DMSO (Figure 3B). Also, the JH-response gene, *Kr-h1*, was induced by hydroprene in larvae and JH III in cells (Figure 3B). These data suggest that JH suppresses HDAC11 gene expression. To determine whether or not JH suppression of the HDAC11 gene requires the JH receptor, Met, *T. castaneum* last instar larvae were injected with dsMet or dsmalE and then treated with hydroprene or cyclohexane. As shown in Figure 3C, dsMet injected larvae showed significantly lower levels of Met mRNA when compared to those in larvae injected with dsmalE. As expected, the HDAC11 mRNA levels decreased in dsmalE injected larvae treated with hydroprene but not in dsMet injected larvae treated with hydroprene. Also, the *Kr-h1* gene was induced in dsmalE injected larvae but not in dsMet injected larvae. These data suggest that Met is required for JH suppression of HDAC11 gene expression.

### Identification of Genes Affected by HDAC11 Knockdown

**FIGURE 4:**
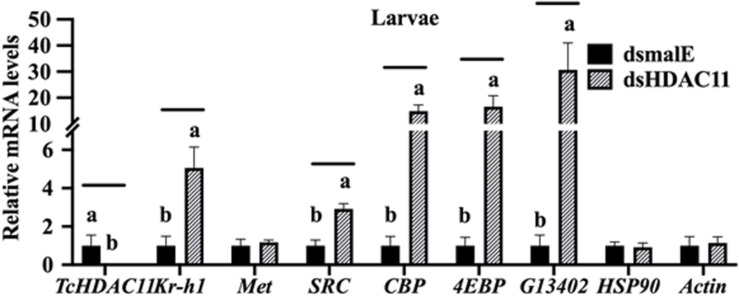
*HDAC11* knockdown in the last instar larvae of *T. castaneum* affects the expression of genes involved in JH action and response. The knockdown of *HDAC11* in newly molted last instar larvae caused an upregulation of genes involved in JH action (*SRC*, *CBP*) and JH response (*Kr-h1*, *4EBP*, *G13402)*. dsHDAC11 or dsmalE was injected into newly molted last instar larvae. Total RNA was extracted at 12 h after treatment, and the mRNA levels of JH-response genes (*Kr-h1*, *4EBP*, *G13402*), genes involved in JH action (*Met*, *SRC*, *CBP*), *HSP90* and *Actin* were quantified. Mean ± SE (*n* = 4) are shown. Means marked with different letters are significantly different from each other, *P* ≤ 0.05 by ANOVA.

The effect of Knockdown of HDAC11 on the expression of JH response genes in larvae were tested using RT-qPCR. *T. castaneum* last instar larvae were injected with dsHDAC11 or dsmalE A, the total RNA isolated from these larvae was used to determine mRNA levels of HDAC11 and genes known to be involved in JH-response. The mRNA levels of HDAC11 decreased and those of three JH-response genes (*Kr-h1*, *4EBP*, *G13402*), SRC, and CBP increased significantly in dsHDAC11 injected larvae when compared to those in larvae injected with dsmalE (Figure 4). However, the expression of Met and two housekeeping genes, Actin and HSP90, were not affected by *HDAC11* knockdown (Figure 4). These data suggest that HDAC11 may influence the expression of JH-response as well as genes coding for proteins involved in JH action.

**FIGURE 5:**
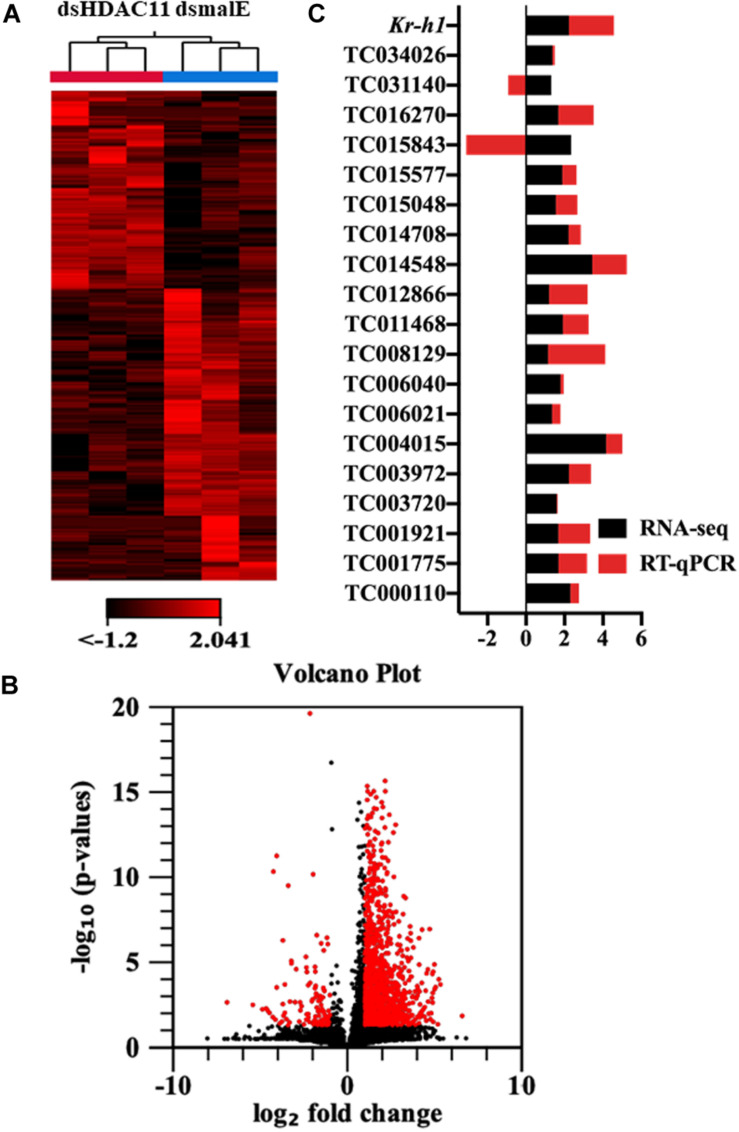
*HDAC11* knockdown in the last instar larvae of *T. castaneum* affects the transcription of genes involved in multiple pathways. **(A)** The heatmap of differentially expressed genes in dsHDAC11 and dsmalE treated insects. **(B)** Differentially expressed genes identified after *HDAC11* knockdown represented as the volcano plot. The X and Y-axis represent the −log_10_ *P*-values and log_2_ fold change of mean normalized values, respectively. The red dots indicate the genes that showed a ≥2-fold difference in expression with a *P* ≤ 0.05. **(C)** RNA-seq data is verified and compared with RT-qPCR represented as a stacked bar graph. RT-qPCR confirmed the expression of 20 selected genes from the up-regulated group (Supplementary Data Sheet_RNA-seq data).

**FIGURE 6:**
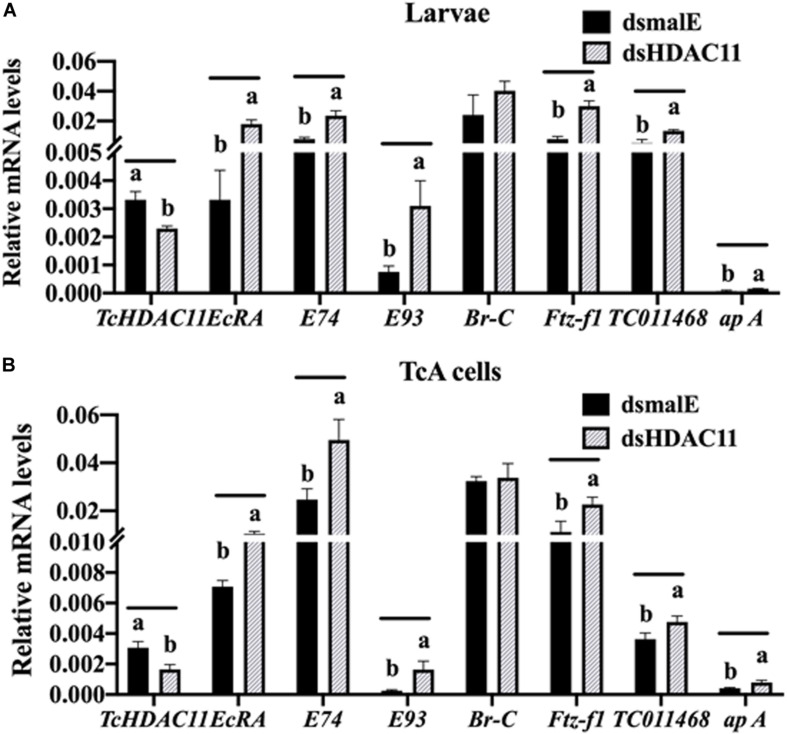
RT-qPCR validation of RNA-seq data in *Tribolium* larvae and TcA cells. **(A)** Differentially expressed genes predicted by RNA-seq analysis are verified by RT-qPCR. Total RNA was extracted at 12 h after injection was used to quantify mRNA levels. Mean ± SE of four replications is shown. Means marked with different letters are significantly different from each other, *P* ≤ 0.05 by *t*-test. **(B)** TcA cells were treated with dsHDAC11 or dsmalE. Total RNA was extracted 72 h after dsRNA treatment was used to quantify mRNA levels. Mean ± SE of four replications is shown. Means marked with different letters are significantly different from each other, *P* ≤ 0.05 by ANOVA.

The RNA isolated from dsHDAC11 and dsmalE injected larvae at 12 h after treatment were sequenced and assembled into transcriptomes and used to identify other targets of HDAC11. Run summary and total read counts of sequencing output are shown in Supplementary Table S2. A heatmap representing the overall pattern of normalized mean expression values of differentially expressed genes (DEGs) is shown in Figure 5A. The DEGs are shown as a volcano plot with red dots indicating statistically significant genes after the EDGE test between treatment and control (Figure 5B). We identified 1913 DEGs (Supplementary Data Sheet), 95% (1815) of these genes are up-regulated, and 5% (98) are down-regulated. Hormone response genes *Kr-h1*, ecdysone induced protein 74EF, and ecdysone receptor are among the up-regulated genes (Supplementary Data Sheet, dsHDAC11-DEG, Table 1). Web-based GO analysis of differently expressed genes showed enrichment of GO terms for binding, especially nucleic acid and protein binding, biological regulation, pigmentation, and developmental process (Supplementary Figure S2). To confirm the results from RNA-seq analysis, we selected 20 genes based on their predicted function and expression levels and verified by RT-qPCR using the RNA isolated from *T. castaneum* larvae. Eighteen out of 20 genes tested showed a positive correlation in their expression levels determined by the two methods (Figure 5C). RT-qPCR analysis of RNA isolated from larvae (Figure 6A) and TcA cells (Figure 6B) also verified an increase in expression levels of EcRA, E74, E93 Ftz-f1, TC011468 and ap A increase predicted by differential gene expression analysis of transcriptomes of larvae treated with dsHDAC11 or dsmalE. A comparison of up-regulated genes between JH III treated TcA cells (Roy and Palli, 2018), and dsHDAC11 treated larvae identified eleven common genes, including the *Kr-h1* (Table 2). Nine genes that code for proteins containing zinc finger, COG5048 domains found in *Kr-h1* are also up-regulated in *HDAC11* knockdown larvae (Table 3). Since dsHDAC1 (George et al., 2019) and dsHDAC11 induced 100% mortality, we compared DEGs between these two datasets and identified several common genes (Supplementary Data Sheet_dsHDAC1 vs. dsHDAC11). Notably, the expression of CBP was up-regulated by seven-fold in both treatments. Since dsHDAC11 larval phenotypes showed enhanced pigmentation, we searched for genes coding for enzymes involved in melanization in our RNA-seq data. Interestingly, several genes coding for enzymes known to function in melanin biosynthesis were up-regulated in dsHDAC11 injected larvae (Table 4).

**TABLE 1:** Expression changes of hormone-response genes identified in larvae treated with dsHDAC11.

Gene symbol	Gene description	Fold Change^a^	*P*-value^b^
*Kr-h1*	Krüppel homolog 1	4.68	0.00
LOC655028	ecdysone-induced protein 74EF	2.03	0.00
*EcR*	ecdysone receptor	2.18	0.03
Tcjheh-r1	juvenile hormone epoxide hydrolase-like protein 1	28.14	0.01
Tcjheh-r2	juvenile hormone epoxide hydrolase-like protein 2	2.03	0.01
Tcjheh-r5	juvenile hormone epoxide hydrolase-like protein 5	2.40	0.00
LOC661705	Phosphoenol pyruvate carboxykinase [GTP]	3.14	0.00
LOC659239	Krueppel homolog 2	3.87	0.00
TcSRC	nuclear receptor coactivator 1	2.24	0.02
LOC660434	broad-complex core protein isoform 6-like	2.59	0.02
LOC658929	nuclear hormone receptor FTZ-F1	2.03	0.04
*USP*	ultraspiracle nuclear receptor	2.08	0.00
LOC664565	CREB-binding protein	7.73	0.00
LOC660626	Hairy	9.33	0.00
LOC658656	Apterous A	4.67	0.00

*^*a*^Experiment – Fold Change (normalized values). ^*b*^Baggerley’s test: normalized values.*

**TABLE 2:** JH-response genes that showed an increase in expression in dsHDAC11 treated larvae.

Gene symbol	Gene description	JH		dsHDAC11	
			
		Fold change	*P*-value	Fold change	*P*-value
LOC660562	rho GTPase-activating protein 100F	115.45	0.020	29.80	0.002
*Kr-h1*	Kr ppel homolog 1	29.91	0.000	4.68	0.000
LOC662658	4-hydroxyphenylpyruvate dioxygenase	3.46	0.000	4.00	0.000
LOC100141923	sodium-coupled monocarboxylate transporter 2-like	202.63	0.004	3.82	0.014
LOC660154	projectin	13.63	0.030	3.68	0.000
LOC659434	fibroblast growth factor receptor homolog 1	95.55	0.030	3.63	0.000
LOC661127	uncharacterized LOC661127	4.02	0.030	3.38	0.000
LOC107398485	sodium-independent sulfate anion transporter-like	4.28	0.030	3.21	0.004
LOC658154	alpha-2C adrenergic receptor	8.08	0.001	2.90	0.012
LOC100142605	ankyrin-3	2.90	0.008	2.38	0.007
LOC103314138	voltage-dependent T-type calcium channel subunit alpha-1G	6.85	0.020	2.14	0.000

**TABLE 3:** COG5048 domains identified in genes up-regulated in dsHDAC11 treated larvae.

Gene symbol	Gene description	Locus tag	Fold change	*P*-value
LOC658487	Zinc finger protein GLIS2 homolog	TC000326	2.49	0.000
LOC659757	Zinc finger protein Gfi-1	TC031695	6.19	0.000
*Kr-h1*	Krüppel homolog 1	TC012990	4.68	0.000
LOC660309	Krueppel-like factor 8	TC006125	2.23	0.000
*Pho*	Pleiohomeotic	TC015577	3.69	0.000
LOC662411	Zinc finger protein 184	TC015048	2.92	0.040
LOC103313120	Zinc finger protein OZF	N/A	3.99	0.050
LOC103314758	Zinc finger protein ZFP69	TC033495	3.11	0.010
LOC107397979	Gastrula zinc finger protein XlCGF26.1-like	N/A	8.52	0.030

*N/A, not available.*

**TABLE 4:** Genes involved in melanin biosynthesis that showed an increase in their expression in *HDAC11* knockdown larvae.

Gene symbol	Gene description	Locus Tag	Fold Change	*P*-value
*Ddc*	dopa decarboxylase	TC013480	4.46	0.000
*Dat*	dopamine N acetyltransferase	TC008204, Nat	2.50	0.000
*Lac1*	laccase 1	TC000821, TcLac1	2.36	0.000
*Th*	tyrosine hydroxylase	TC002496	3.39	0.000
*Y-2*	yellow-2	TC003539	4.97	0.000

### *HDAC11* Knockdown Increases Acetylation Levels of Histone H3

**FIGURE 7:**
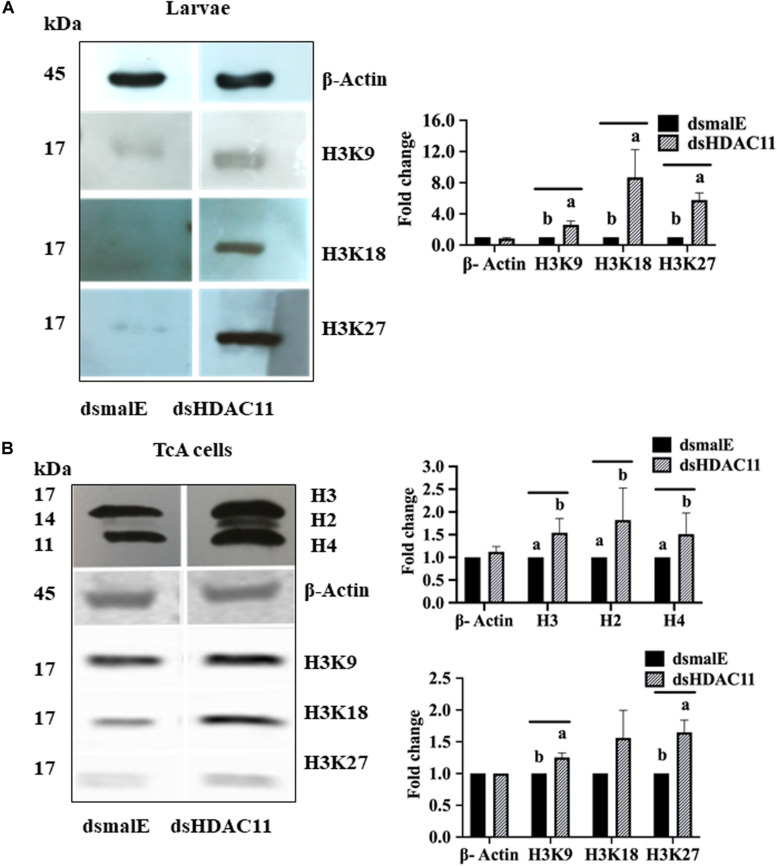
*HDAC11* knockdown affects acetylation levels of histone H3 in *T. castaneum*. **(A)** The total protein extracted from dsHDAC11 or dsmalE injected larvae were resolved on SDS-PAGE gels, transferred to western blots, and probed with antibodies recognizing Acetyl-Histone H3 (Antibody Sampler Kit # 9927-Cell Signaling (H3K9, H3K14, H3K18, H3K27, and H3K56). ß-actin served as a loading control. The HRP-linked IgG (#7074-Cell Signaling) was used as a secondary antibody. Band densities were determined by Image-J software and normalized with loading control protein-ß-Actin. The Mean + SE (*n* = 3) band densities are shown. Means marked with different letters are significantly different from each other, *P* ≤ 0.05 by ANOVA. **(B)** Acetylated-Lysine (Ac-K^2^-100) MultiMab^TM^ Rabbit mAb mix #9814 was used to detect acetylation levels of proteins extracted from TcA cells exposed to dsHDAC11 or dsmalE. The acetylation levels of histone H3K9, H3K18 and H3K27 increased in HDAC11 knockdown cells were detected as described in Figure 7A. The band densities were quantified and plotted as described in Figure 7A. The Mean + SE (*n* = 3) band densities are shown. Means marked with different letters are significantly different from each other, *P* ≤ 0.05 by ANOVA.

Increase in acetylation levels of H3K9, H3K18, and H3K27 was detected in dsHDAC11 treated larvae compared to the levels in dsmalE treated larvae (Figure 7A). Also, TcA cells exposed to dsHDAC11 showed an increase in acetylation levels of core histones H3, H2, and H4 (Figure 7B) compared with control cells treated with dsmalE. An increase in acetylation levels of H3K9, H3K18 and H3K27 was detected in dsHDAC11 treated cells compared to control cells treated with dsmalE (Figure 7B). These data suggest that H3 is one of the targets for HDAC11 deacetylation.

## Discussion

We recently reported on the function of class I HDACs in JH suppression of metamorphosis in *T. castaneum* (George et al., 2019). Here, we investigated the role of the sole member of class IV HDACs, HDAC11. HDAC11 knockdown induced complete lethality during the larval stage. Human HDAC11 is biochemically distinct from other HDACs but phylogenetically closely related to class I HDACs (De Ruijter et al., 2003). Similarly, phylogenetic analysis of HDACs in *T. castaneum* revealed that TcHDAC11 is close to class I than to class II deacetylases (Figure 1B).

Interestingly, we observed that knockdown of the *HDAC11* gene during the final instar larval stage of the red flour beetle, *T. castaneum* resulted in a dark-colored larval phenotype that eventually died. Human HDAC11 is reported to be involved in the regulation of different inflammatory responses and diverse immune functions (Yanginlar and Logie, 2018). Dopa decarboxylase (Ddc) and phenoloxidase (PO) are necessary for insect cuticular melanization, and the molecular action of 20-hydroxyecdysone on various transcription factors leads to Ddc expression in *Manduca sexta* (Hiruma and Riddiford, 2009). Strict regulation of immune response and melanization is crucial for the proper development and survival of insects. RNA-seq data showed that the genes coding for enzymes (Tyrosine hydrolase, *Ddc*, Yellow-2, Laccase 1, Dopamine N acetyltransferase) known to be involved in insect melanin biosynthesis are up-regulated in HDAC11 knockdown larvae (Table 4). In *D. melanogaster*, microbial infection triggers activation of phenoloxidases; serine proteases and serine protease inhibitors (serpins) control the sites of infection (Tang, 2009). Differential expression of genes coding for several serine proteases, toll-like receptors, and serpins were observed in HDAC11 knockdown samples (Datasheet_melanization terms). Melanization and toll pathway share similar serine proteases in *D. melanogaster* (Dudzic et al., 2019).

Inhibition of HDAC11 induced the expression of p53 in human liver cancer cells and promoted apoptosis (Gong et al., 2019). HDAC11 is reported to be overexpressed in several carcinomas, and HDAC11 depletion causes cell death and inhibits metabolic activity in controlling proliferation in several human carcinoma cell lines (colon, prostate, ovarian cell lines) (Deubzer et al., 2013). Similarly, HDAC11 depletion in human neuroblastoma cells triggers caspase activation and caspase-dependent apoptosis (Thole et al., 2017). HDAC11 depletion in MYCN-driven neuroblastoma cell lines strongly induces cell death, mostly mediated by apoptotic programs (Thole et al., 2017). Inhibitor studies in mouse models showed that HDAC11 plays an important role in oncogene-induced hematopoiesis in myeloproliferative neoplasms (MPNs) (Yue et al., 2020). Based on these previous findings, it is tempting to hypothesize that HDAC11 knockdown may induce the death of some internal tissues resulting in the dark color detected in larvae. However, further work is required to identify specific tissues/cells involved and to uncover mechanisms behind the phenotype detected after HDAC11 knockdown in *T. castaneum* larvae.

Another finding of this research is the discovery that HDAC11 is required for larval development and metamorphosis in *T. castaneum*. This is the first report on the role of HDAC11 on the growth and development of insect larvae. RNA sequencing and RT-qPCR analysis showed that knockdown in HDAC11 affects the expression of JH and 20E response genes as well as those coding for proteins involved in the action of these two hormones. Since both JH and 20E play critical roles in larval development and metamorphosis (Jindra et al., 2013); HDAC11 may block larval development and metamorphosis by affecting the action of these two hormones. RNA-seq analysis revealed several genes, including transcription factor sox-10, Hairy, CBP, SRC, chromatin remodeler SWI/SNF complex subunit SMARCC2, polycomb complex protein BMI-1-A, polycomb protein Scm, BTB/POZ, ultrabithorax, EcR, tramtrack, ecdysone-induced protein 74EF, and nuclear hormone receptor FTZ-F1 that showed an increase in expression in HDAC11 knockdown larvae. These data suggest that the deacetylation of histones and other proteins by HDAC11 may lead to a decrease in expression of these genes involved in JH and 20E action and response. However, the precise mechanisms involved in the arrest in larval development induced by HDAC11 knockdown remains to be elucidated. Further studies are required to uncover molecular mechanisms governing these phenotypes.

Apterous A (ap A, TC003972) and mortality factor 4-like protein 1 (TC011468) are significantly up-regulated in dsHDAC11 treated larvae. RT-qPCR reconfirmed RNA-seq predictions (Figures 6A,B). In *D. melanogaster*, apterous encodes a member of LIM (*Lin11*, *Isl-1& Mec-3*) homeobox transcription factor which contributes to the identity of wing cells, JH production, and neuronal pathfinding (Cohen et al., 1992). Mortality factor 4-like protein 1 (TC011468), also known as NuA4 complex subunit EAF3 homolog-like protein is a MORF-related gene and part of the Tip60 chromatin remodeling complex in *D. melanogaster*. Tip60 is involved in DNA repair by acetylating phosphorylated H2AV in *D. melanogaster* (Kusch et al., 2004). Mortality factor 4 like 1 protein regulates chromatin remodeling and mediates epithelial cell death in a mouse model of pneumonia (Zou et al., 2015). Interestingly, HDAC inhibitors activity leads to the modulation of expression of various genes and in turn, induces growth arrest, differentiation, and apoptotic cell death (Marks et al., 2000). Moreover, HDAC11 overexpression inhibits the cell cycle progression in fibroblast of Balb/c-3T3 cells. Also, the HDAC11 transcript was identified as a platelet-derived growth factor (PDGF) target, and HDAC11 mRNA abundance correlates inversely with proliferative status (Bagui et al., 2013). We identified fibroblast growth factor receptor homolog 1, ankyrin-3 as common genes up-regulated in HDAC11 knockdown larvae, or JH III treated cells (Table 2). Nuclear hormone receptors and their transcriptional coregulators were expressed in neural stem cells, and their expression was altered during differentiation induced by fibroblast growth factor 2 (FGF2) withdrawal. FGF2 withdrawal strongly induced the mRNA expression of HDAC11 in mouse cells (Androutsellis-Theotokis et al., 2013). Chromatin modifier, HDAC11, regulates lymph node metastasis development and dissemination in the breast cancer experimental model (Leslie et al., 2019). Human HDAC11 expression is limited to kidney, heart, brain, skeletal muscle, and testis, suggesting a tissue-specific function (Gao et al., 2002). Small interfering RNAs (siRNAs) that selectively inhibited HDAC11 expression, significantly up-regulated OX40L, and induced apoptosis in Hodgkin lymphoma (HL) cell lines. Silencing HDAC11 increased the production of tumor necrosis-α (TNF-α) and IL-17 in the supernatants of HL cells. An HDAC inhibitor study in human cell lines revealed that HDAC11 plays an essential role in regulating OX40 ligand expression in Hodgkin lymphoma (Buglio et al., 2011). The protein “Eiger” is the close homolog for OX40L in *T. castaneum* and *D. melanogaster* eiger (egr) encodes the TNF superfamily ligand that activates the intracellular JNK pathway, which mediates cell death, tumor suppression, and growth regulation (Igaki et al., 2002; Shklover et al., 2015). Our studies revealed that HDAC11 is essential for survival, and RNAi mediated knockdown induce developmental arrest and mortality.

**FIGURE 8:**
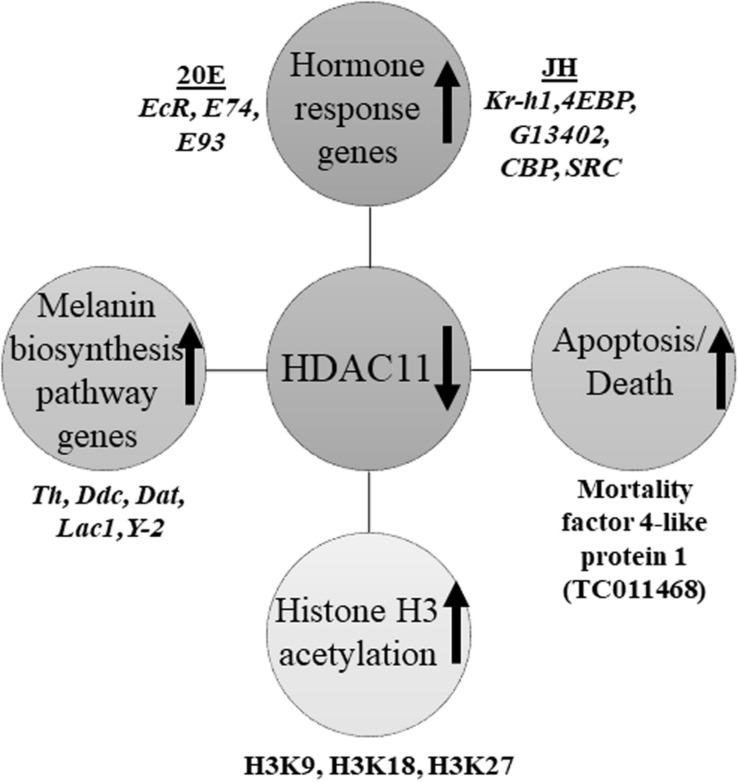
Schematic representation of HDAC11 action in the red flour beetle. Here we summarized the results RNA sequencing and RT-qPCR analysis showed that knockdown in HDAC11 affects the expression of JH and 20E response genes as well as those coding for proteins involved in the action of these two hormones. Since both JH and 20E play critical roles in larval development and metamorphosis, HDAC11 may have blocked larval development and metamorphosis by affecting the action of these two hormones. The GO terms for pigmentation is significantly enriched in HDAC11 knockdown samples compared to the control. RNA-seq data showed that the genes coding for enzymes (Tyrosine hydrolase, *Ddc*, Yellow-2, Laccase 1, Dopamine N acetyltransferase) involved in insect melanin biosynthesis are up-regulated. RNAi mediated knockdown of HDAC11 induces mortality in larvae, pupae, and adults. Mortality factor 4-like protein 1 (TC011468) and apterous A (TC003972) were identified as differentially expressed genes in HDAC11 knockdown larvae. HDAC11 knockdown resulted in an increase in acetylation levels of histones H3, especially H3K18 and H3K27.

HDAC11 regulates oligodendrocyte-specific gene expression and cell development in the cell line of rats by deacetylation of histone H3K9/K14 (Liu et al., 2009). Our western blots analysis showed that HDAC11 regulates acetylation levels of H3, specifically H3K9, H3K18 and H3K27. As demonstrated for HDAC1 in *T. castaneum* (George et al., 2019), HDAC11 may also influence acetylation levels of histones, especially H3, and regulate promoter access and, consequently, the expression of genes involved in JH and 20E action and response. In conclusion, we showed that HDAC11 knockdown affects hormone action and melanin biosynthesis and thereby arrest in development and metamorphosis of the red flour beetle, *T. castaneum* (Figure 8).

## Data Availability Statement

We have deposited the short-read (Illumina HiSeq 4000) sequence data in the NCBI SRA (accession numbers PRJNA495026 and PRJNA612004). BioSample metadata are available in the NCBI BioSample database (http://www.ncbi.nlm.nih.gov/biosample/) under accession numbers SAMN10203356, SAMN10203357, SAMN14356366, and SAMN14356367).

## Author Contributions

SG and SP designed the experiments, and wrote the manuscript. SG carried out the experiments. Both authors contributed to the article and approved the submitted version.

## Conflict of Interest

The authors declare that the research was conducted in the absence of any commercial or financial relationships that could be construed as a potential conflict of interest.
