# Supplementary material for: Histone Deacetylase 11 Knockdown Blocks Larval Development and Metamorphosis in the Red Flour Beetle, Tribolium castaneum
Source: Front Genet. 2020 Jul 3;11:683. doi: 10.3389/fgene.2020.00683 (PMC7348043; doi:10.3389/fgene.2020.00683)
Supplement: Supplementary file 1 [file Presentation_1.PDF]

Supplementary Information

Histone deacetylase 11 Knockdown Blocks Larval Development and Metamorphosis in the Red Flour Beetle, *Tribolium castaneum*

Smitha George and Subba Reddy Palli*

Department of Entomology, University of Kentucky, Lexington, Kentucky 40546, USA

*Corresponding author

Phone: 859-257-4962

Fax: 859-323-1120

Email: rpalli@email.uky.edu

```
Dm_HDAC11       MKRLTERVHHEEQESEEVVWVKRDDARSAGKLPIVFSRNYAVRFGGLERLHPFDAAKGKH      60
Tc_HDAC11       ------------------MNTKLYVDIGPEQWPIIYRPEYNVRFLGLEKLHPFDAGKWGN      42
Hs_HDAC11       ----------------MLHTTQLYQHVPETRWPIVYSPRYNITFMGLEKLHPFDAGKWGK      44
                                    .:        : **::  .* : * ***:******.*  :

Dm_HDAC11       IHKLLCAQLQLDDGSFYEPTELTKDQLRRIHTREYLKSLRWSMNVACIAEVPLMAFVPNR      120
Tc_HDAC11       IYKYLKSCGLVNDETLSVPNEATTEDLLTVHTKKYLKSLKCSFNVALIAEVLPLCLVPNY      102
Hs_HDAC11       VINFLKEEKLLSDSMLVEAREASEEDLLVVHTRRYLNELKWSFAVATITEIPPVIFLPNF      104
                : : *     :.*  :    * : ::*  :**:.**:.*: *: ** *:*:  : ::**

Dm_HDAC11       YIQRSYLRPMRFQAAGSILAGKLALDYGWAINLGGGFHHCCSYRGGGFCPYADISLLIVR      180
Tc_HDAC11       LVQRGYLRPMRFQTGGSVLAGKLALERGWAINIGGGFHHCCGSKGGGFCVYADITLLIHF      162
Hs_HDAC11       LVQRKVLRPLRTQTGGTIMAGKLAVERGWAINVGGGFHHCSSDRGGGFCAYADITLAIKF      164
                 :**  ***:* *:.*:::*****:: *****:*******.. :***** ****:* *

Dm_HDAC11       LFEQEPFRVRRIMIVDLDAHQGNGHERDFNNVAAVYIFDMYNAFVYPRDHVAKESIRCAV      240
Tc_HDAC11       VFNHHPRSVQNVMIVDLDAHQGNGYQRDFKDNPNVYIIDVYNKGIYPFDKLAKEYITRKV      222
Hs_HDAC11       LFERVE-GISRATIIDLDAHQGNGHERDFMDDKRVYIMDVYNRHIYPGDRFAKQAIRRKV      223
                :*::    : .  *:*********::*** :   ***:*:**  :** *:.**: *   *

Dm_HDAC11       ELRNYTEDGFYLRQLKRCLMQSLAEFRPDMVVYNAGTDVLEGDPLGNLAISAEGVIERDR      300
Tc_HDAC11       ELAHFTEDDEYLDKVAKNLTEALAEFCPQLIVYNAGTDILKGDALGCLSVSPQGIIERDE      282
Hs_HDAC11       ELEWGTEDDEYLDKVERNIKKSLQEHLPDVVVYNAGTDILEGDRLGGLSISPAGIVKRDE      283
                **   ***. ** :: : : ::* *. *:::*******:*:** ** *::*  *:::**.

Dm_HDAC11       LVFSTFRALGIPVVMLLSGGYLKASAGVITDSIVNLRLQGLLN-----------------      343
Tc_HDAC11       LVFREARSRNIPIVMLTSGGYLKKSAKIIATSIKNLHDSGLITGPQLRY-----------      331
Hs_HDAC11       LVFRMVRGRRVPILMVTSGGYQKRTARIIADSILNLFGLGLIGPESPSVSAQNSDTPLLP      343
                ***   *.  :*::*: **** * :* :*: ** **   **:

Dm_HDAC11       ----      343
Tc_HDAC11       ----      331
Hs_HDAC11       PAVP      347
```

Figure S1: HDAC11 sequence conservation in *Drosophila melanogaster* (Dm), *Tribolium castaneum* (Tc) and *Homo sapiens* (Hs). Comparison of *Tribolium castaneum* full-length HDAC11 amino acid sequence with *D. melanogaster* and *H. sapiens* HDAC11 amino acid sequences by Clustal2.1 (Raghava and Barton, 2006).

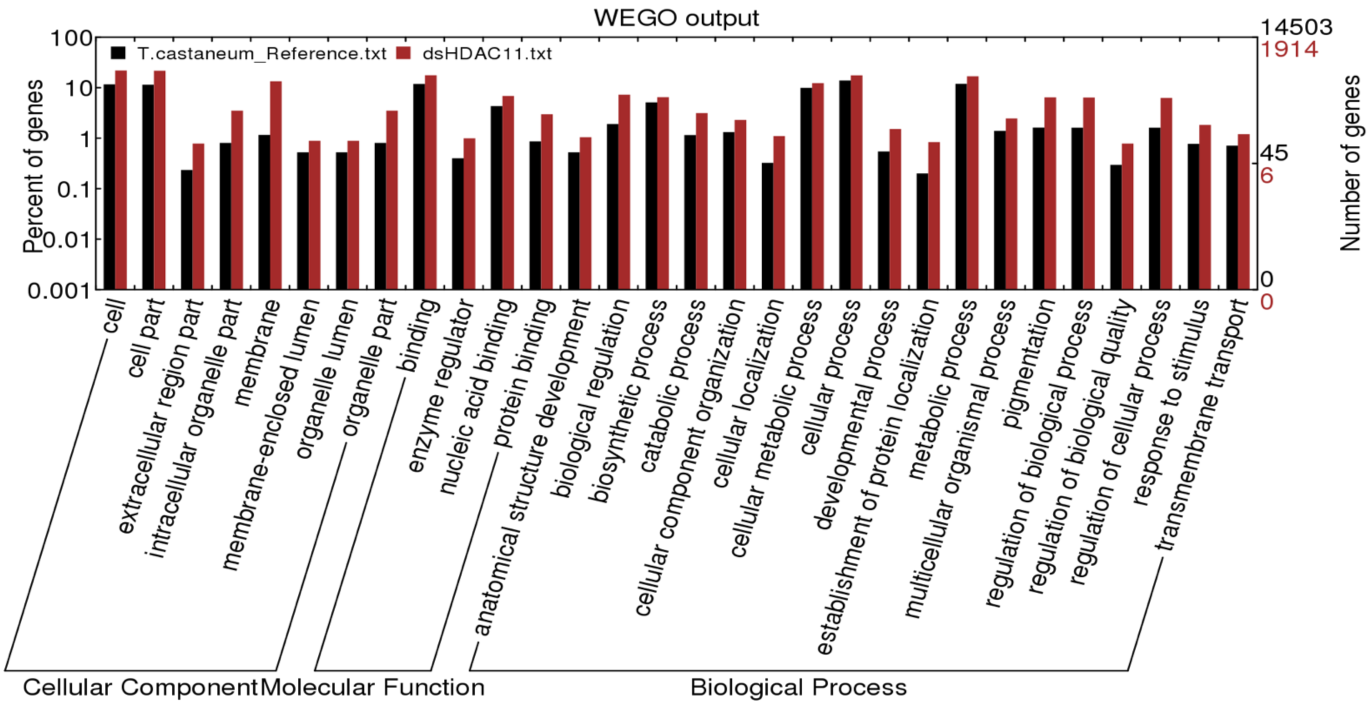

Figure S2: WEGO

The WEGO analysis identified enrichment of nucleic acid binding, developmental process, pigmentation, and regulation if biological process. The WEGO histogram shows the number and percent of genes in the cellular component, molecular component, and biological function ontology classification group for *T. castaneum* transcriptome and 1913 differentially expressed genes in *HDAC11* knockdown larvae. The Blast2GO PRO-plugin software was used for the functional annotation of the transcripts.

Table S1: List of dsRNA and RT-qPCR primers used in this study.

Gene	Primer
TcdsHDAC11F	TAATACGACTCACTATAGGGATTGTTGCGGTAGCAAAGGTGGTG
TcdsHDAC11R	TAATACGACTCACTATAGGGTCGTCTTCGGTAAAGTGTGCCAGT
TcqHDAC11F	CACACTTTACCGAAGACGACGAGT
TcqHDAC11R	AGTTCATCCCGTTCGATGATTCCC
TcdsHDAC11-2F	TAATACGACTCACTATAG GG GGTGCCAAATGAAGCAACAA
TcdsHDAC11-2R	TAATACGACTCACTATAG GG ACGTTCCAGAGCTAACTTTCC
Tcq011468F	GTTTGCAGTTGGGACGAATG
Tcq011468R	TTCGTCTTGCTGGGTTGAG
Tcq003972F	GTGAGGAGTGATAGCGTTGTT
Tcq003972R	AGAGACAGGGTGTCCATAGAG
TcqHSP90F	GCGCTAAGTGAAGAGCTAAGA
TcqHSP90R	ATGCACACACGAACAAATCAC
Tcq4EBPF	ATCACCGATGGCAAGACAAGTGAC
Tcq4EBPR	ATGGCAGTTCAGAAGGGTCGTTGA
TcqG13402F	ACTGTGCCGAGTTTAGGA
TcqG13402R	GAATGCCAGTGGGTCAGG
TcqCBPF	GGTCCCGATGGTAAGAAGAAAG
TcqCBPR	CCGAGAGATCATTACCCGTTTG
TcqE93F	CTCTCGAAAACTCGGTTCTAAACA
TcqE93R	TTTGGGTTTGGGTGCTGCCGAATT

RP49, EcRA, E74, Ftz-f1, Br-C (Tan and Palli, 2008), SRC, Met, Kr-h1 (Zhang et al., 2011) were reported previously.

Table S2: Summary statistics of RNA-sequencing output of dsHDAC11

A. Run Summary

**Lane**	**PF* Yield (bp)**	**Number of PF* Clusters****	**Q30%**	**Average Quality Score**
7	15,963,128,520	313,002,520	95.64	39.04

* PF: Passed Filter. ** For single-read sequencing (SR), number of reads = number of clusters.

B. Read count statistics

Samples	Read Count	Single, mapped %	Reverse % of reads mapped
MalE-1	12,545,282	51.11	80.56
MalE-2	16,562,495	45.23	82.45
MalE-3	23,183,581	53.19	82.30
HDAC11-1	12,086,140	46.21	79.14
HDAC11-2	9,267,486	51.73	80.99
HDAC11-3	34,175,140	44.94	75.16

References

Raghava, G.P., and Barton, G.J. (2006). Quantification of the variation in percentage identity for protein sequence alignments. *BMC Bioinformatics* 7**,** 415.

Tan, A., and Palli, S.R. (2008). Ecdysone [corrected] receptor isoforms play distinct roles in controlling molting and metamorphosis in the red flour beetle, *Tribolium castaneum*. *Molecular Cellular Endocrinology* 291**,** 42-49.

Zhang, Z.L., Xu, J.J., Sheng, Z.T., Sui, Y.P., and Palli, S.R. (2011). Steroid receptor co-activator is required for juvenile hormone signal transduction through a bHLH-PAS transcription factor, Methoprene tolerant. *Journal of Biological Chemistry* 286**,** 8437-8447.
